# Medical Wards of the Mercy Hospital

**Published:** 1860-01

**Authors:** 


### CLINICAL REPORTS.

Medical Wards of the Mercy Hospital. Service of Dr. N. S. DAVIS.

Nov. 1st. Male Ward, No. 1. Dr. Davis remarked to the class, that he should occupy their time this morning, chiefly with two cases.

Case 1. *Chronic Ague complicated with sub-acute Duodenitis and Pneumonia.* The substance of the remarks upon this case were as follows: The patient before us is a native of Ireland, aged about 25 years, a laborer, and has been spending some months in the South. Whilst there, he was attacked with periodical fever, and found his way into a Hospital in St. Louis. By judicious treatment his fever was arrested, and in due time he was discharged. Probably, from undue exposure, he soon had a relapse in the form of a tertian intermittent.

Without any regular treatment, the patient has continued to suffer from this disease, in the meantime enduring more or less exposure and fatigue, until he reached this city, and was admitted into the Hospital yesterday. You see, at a glance, that his skin and conjunctival membrane of the eyes present a deep yellow color; his pro-labia are pale; tongue coated with a yellowish white fur; and his general aspect that of anæmia. His skin his only slightly above the natural temperature; pulse 90 per minute and soft; bowels inactive; respiration rather short, but not difficult; moderate cough, with an acute, sore pain in the left sub-axillary region ; urine scanty and very high colored. It is evident that the patient has been laboring under the intermittent fever long enough to induce considerable diminution of the red corpuscles in the blood, as is shown by the paleness of his lips and the general muscular weakness; but this does not satisfactorily explain either the pain in the left side of the chest, or the jaundiced hue of the skin, with a sense of fullness and soreness in the epigastric and right hypochondriac regions. To determine the origin of these symptoms, we must resort to auscultation and percussion, or in other words, to a physical exploration of the chest and abdomen.

Uncovering the patient for this purpose, you see the epigastric and right hypochondriac regions somewhat more full than natural, but as we percuss, you learn from the tympanitie resonance, that most of the fullness is from gaseous distension of the intestines; while the hepatic dullness is restricted to its natural limits. The patient complains, however, that the percussion causes a sore pain over most of the hepatic region. From the tenderness and fullness of the right hypochondriac and epigastric regions, it is evident that a low grade of inflammation exists in the liver, and probably also in the mucous membrane of the duodenum, which would fully explain the jaundiced hue of the patient. Finding nothing unnatural in the left hypochondric region, we will pass to an examination of the chest. As we percuss extensively over its surface, you detect no unnatural sounds, until we come to the sub-axillary region of the left side. Here the resonance is diminished, indicating that the parts within are more dense than natural. If you will take each his turn in listening through the stethescope applied to this region, you will hear distinctly a fine crepitant rale, indicative of pneumonic inflammation in the early stage of its progress. We are now prepared to explain all the symptoms that the case presents. We have a chronic or protracted intermittent, complicated with a low grade of hepatic and duodenal inflammation, by which the digestive function is impaired, the hepatic ducts obstructed, and the coloring matter of the bile retained in the blood to such an extent as to stain all the tissues a yellow color; while a more acute grade of inflammation has invaded the lower lobe of the left long.

The indications for the treatment are three, namely: the interruption of the intermittent paroxyms; the removal of the local inflammations; and the restoration of the blood and tissues to their normal conditions. The time has been, when the detection of a local inflammation in connection with a periodical fever would cause the first of these indications to be superceded by the second, under the idea that the tonic qualities of the quinine rendered its exhibition unsafe, while local inflammation existed in any of the textures of the body. Experience, however, has fully demonstrated the fallacy of this idea, and shown that the prompt interruption of the febrile exacerbations by quinine, actually facilitates the reduction of the local inflammation. Hence, we shall endeavor to fulfill, in this case, both the first and second indications by the following remedies:

| | | |
|---|---|---|
| ℞ | Sulph. Quinine, | 12 grs. |
| | Proto-Chloride Hydrarg. | 12 " |
| | Pulv. Opii, | 6 " |

Mix and divide into four powders. Give one every four hours. Also between the powders, give a teaspoonful of the following mixture, viz:

| | | |
|---|---|---|
| ℞ Hive Syrup, | ℥ j. | |
| Tinct. Bloodroot, | ℥ ss. | |
| Tinct. Opii et Camph. | ℥ jss. | |
| Tinct. Verat Viride, | ʒ j. | Mix. |

To-morrow, after all the powders have been taken, the bowels should be moved by a dose of castor oil; after which, a powder composed of quinine, 2 grs., nit. potassa, 5 grs., and pulv. opii, 1 gr., may be given every four hours, and a blister plaster applied to the lower part of the left side of the chest. These means will probably prove sufficient to interrupt the intermittent paroxysms, and completely remove the pneumonic inflammation in two or three days; leaving only the general debility, with more or less duodeno-hepatic derangement for further treatment. If so, we shall direct the following pills, which we have often seen effectual in similar cases, viz:

| | |
|---|---|
| ℞ Ext. Cornus Florida, | ʒ j. |
| Sulphas Ferri, | 30 grs. |
| Blue Mass, | 10 " |
| Ext. Taraxicum, | 30 " |

Mix and divide into thirty pills; one to be given before each meal, and at bed-time. But as you will have an opportunity to see the progress of the case from time to time, we will leave it for the present, and pass to an adjoining bed.

Case. 2. This patient is a native of Ireland, aged about 40 years, a laborer. He has been admitted to the Hospital since our visit yesterday. You see his countenance is expressive of anxiety and severe suffering, and he tells us that about three days since he was attacked with severe pain in the abdomen, which still continues, and is coupled with extreme tenderness over the whole epigastric and umbilical regions. His urine is scanty; his bowels quiet; considerable thirst, with a disposition to reject drinks by vomiting; pulse soft, and not more than 90 per minute. All the symptoms in this case point to the abdomen as containing the seat of disease, while the acute pain and tenderness would equally indicate its inflammatory nature. We may find severe pain in the abdomen from colic; but this, instead of being accompanied by acute tenderness, is generally relieved by pressure. We may also find severe pain in the abdomen from strangulated hernia, either concealed or manifest, or from intussusception. But in either of these conditions the pain would be more circumscribed, that is, referred to some particular part of the abdomen, and be accompanied by complete obstruction of the bowels. Again, in either of the last conditions named, before three days had elapsed, as in this case, the vomiting would be frequent and perhaps stercoraceous with great general prostration. In the patient before us, however, the pain and tenderness are both diffused, the vomiting is not persistent, and free fecal evacuations have occurred since the attack commenced. Hence, we regard it as hardly possible that the present case is one of intestinal obstruction or strangulation. We should regard this as a case of sub-acute inflammation of the peritoneal covering of the intestines. If it involved that part of the peritoneum lining the abdominal parieties, there would be a much greater degree of tenseness and fullness of the abdomen, and if it extended to the mucous membrane, there would be diarrhœa. The consequences of peritoneal inflammation, when uncontrolled, are thickening of the membrane, plastic exudations, and serous effusion. The second often leads to adhesions, and the third to ascites or abdominal dropsy. Most pathologists, in treating of the nature of inflammation, have restricted their attention too exclusively to the condition and movements of the blood or fluids in the part affected. Thus, Dr. Williams makes inflammation consist, essentially, of a determination of blood to the structure involved, with the circulation through it partly increased and partly diminished. We regard every inflammation as involving *three* primary elements or morbid conditions, namely: an accumulation of blood in the part, an exaltation of that elementary property of the tissue which we call susceptibility; and an alteration of the vital affinity.

If the accumulation of blood in the part is accompanied by an active determination to it, with increase of both the susceptibility and affinity, it constitutes what is familiarly known as active, sthenic, or phlegmonous inflammation. If, on the other hand, the accumulation of blood in the part results not from increased determination, but from an impaired action of the capillaries themselves, with dimunition of vital affinity, while susceptibility alone is increased, it constitutes asthenic or aplastic inflammation.

We thus claim that the movements of the fluids and the properties of the solids are both necessarily involved in every true inflammatory process. Hence, we have two uniform and rational indications for treatment, namely, to allay the morbid susceptibility, and to diminish the fullness of blood in the part. Anodynes and the local application of cold, constitute the principal means for accomplishing the first: while the means of accomplishing the second will depend upon the immediate cause of accumulation. Thus, where active determination of blood to the part inflamed, exists, depletion and arterial sedatives will be required; but if the cause of the accumulation is an impaired condition of the capillaries of the part, then, instead of sedatives, such stimulants or excitants as are capable of giving increased tone and contractility to the capillary system, will be most promptly efficient. These observations relate to inflammation in its first stage or elementary condition. If it has existed long enough to produce secondary effects, such as infiltration of texture; effusions, either serous or sanguine; softening, suppuration, &c., these will afford other indications for remedial agencies. In the case before us, there is not that fullness of pulse, or force in the action of the heart, which would call for either depletion or sedatives; neither are there any signs of effusion. Hence the only clear indications are to subdue the extreme morbid susceptibility of the inflamed membrane, and overcome the irritability of the stomach.

The most efficient remedies we possess for this purpose, are narcotic fomentations and full doses of opium, with alterative doses of calomel. To be effectual in such cases, the opium must be given in doses sufficient, not only to allay the pain, but to induce more or less sleep. In inflammations of the serous membranes, this can be done with impunity. But when the respiratory organs are involved, causing increased secretion into the air passages, narcotism, by suspending cough, and efforts to clear away the excessive secretion, greatly increases the danger of suffocation. It is necessary to remember this, especially when prescribing for children. For the patient now before us, we will direct fomentations of hops or aconite leaves over the abdomen, and give a powder composed of pulv. opii. 2 grs, and calomel 2 grs, every two hours, until six doses are taken, unless the patient sooner becomes easy, and exhibits a disposition to sleep. If this should occur the interval between the doses should be lengthened to four hours.

We add the calomel to the opium, in such cases, partly to lessen the gastric irritability, and partly to keep up those important secretory actions which the opium alone would retard or entirely suspend. If we can succeed in bringing the patient readily under the influence of these remedies, the inflammatory process will rapidly abate, and at the end of 36 hours we may suspend their use, and cause a mild but efficient movement of the bowels. But as the clinique hour has already expired, we must omit further remarks until you visit the Ward again.

